# Electrocardiographic ST-segment elevation with prominent R waves in precordial leads

**DOI:** 10.1007/s12471-022-01692-w

**Published:** 2022-05-10

**Authors:** A. Y. Andreou, A. R. Pérez-Riera

**Affiliations:** 1 Department of Cardiology, Limassol General Hospital, Limassol, Cyprus; 2 University of Nicosia Medical School, Nicosia, Cyprus; 3 Centro Universitario Saúde ABC, Santo André, SP Brazil; 4 Uninove University campus Mauá, São Paulo, Brazil

A 79-year-old man with a history suspicious of vasospastic angina presented after he had experienced angina at rest of approximately 10 min, which resolved spontaneously. Electrocardiography (ECG) recorded on admission—when he had no angina—showed atrial fibrillation, right bundle branch block and negative T waves of ischaemia in leads V2–V3 (Fig. 1). While the patient was still in the emergency department, he experienced another episode of angina, during which the ECG depicted in Fig. 2 was recorded. His angina resolved promptly following treatment with sublingual nitroglycerin, while the ECG changes returned to the pre-angina baseline state. Serum troponin I level was normal (< 0.5 ng/ml).

**Fig. 1:**
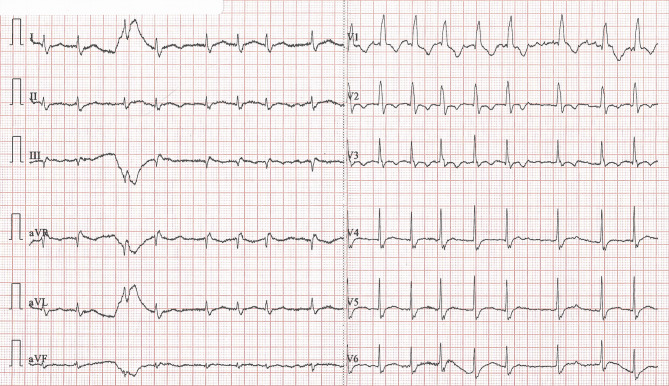
Electrocardiogram on admission when patient was pain free

**Fig. 2:**
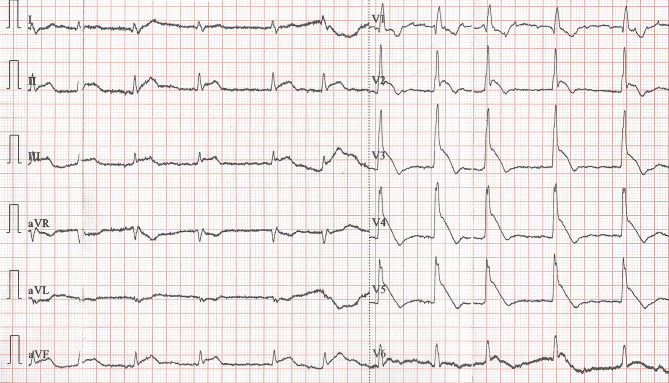
Electrocardiogram when patient experienced ongoing angina

What is your diagnosis, and what could be the cause of the ECG abnormalities during the anginal episode?

## Answer

You will find the answer elsewhere in this issue.

